# Alcohol Intake as a Risk Factor for Acute Stroke: The INTERSTROKE Study

**DOI:** 10.1212/WNL.0000000000201388

**Published:** 2023-01-10

**Authors:** Andrew Smyth, Martin O'Donnell, Sumathy Rangarajan, Graeme J. Hankey, Shahram Oveisgharan, Michelle Canavan, Clodagh McDermott, Denis Xavier, Hongye Zhang, Albertino Damasceno, Alvaro Avezum, Nana Pogosova, Aytekin Oguz, Danuta Ryglewicz, Helle Klingenberg Iversen, Fernando Lanas, Annika Rosengren, Salim Yusuf, Peter Langhorne, on behalf of the INTERSTROKE Investigators

**Affiliations:** From the Population Health Research Institute (A.S., M.O.D., S.R., S.Y.), McMaster University and Hamilton Health Sciences, ON, Canada; HRB Clinical Research Facility Galway (A.S., M.O.D., M.C., C.M.), Department of Medicine, University of Galway, Ireland; Medical School (G.J.H.), Faculty of Health and Medical Sciences, The University of Western Australia, Perth, Australia; Rush Alzheimer Disease Research Center (S.O.), Rush University Medical Center, Chicago, IL; St John's Medical College and Research Institute (D.X.), Bangalore, India; Beijing Hypertension League Institute (H.Z.), China; Faculty of Medicine (A.D.), Eduardo Mondlane University, Maputo, Mozambique; Hospital Alemao Oswaldo Cruz (A.A.), Sao Paulo, Brazil; National Medical Research Center of Cardiology (N.P.), Moscow, Russia; Department of Internal Medicine (A.O.), Faculty of Medicine, Istanbul Medeniyet University, Turkey; Military Institute of Aviation Medicine (D.R.), Warsaw, Poland; Stroke Center (H.K.I.), Department of Neurology, Rigshospitalet, University of Copenhagen, Denmark; Faculty of Medicine (F.L.), Universidad de La Frontera, Temuco, Chile; Sahlgrenska University Hospital and Sahlgrenska Academy (A.R.), University of Gothenburg, Sweden; and Academic Section of Geriatric Medicine (P.L.), Glasgow Royal Infirmary, University of Glasgow, United Kingdom.

## Abstract

**Background and Objectives:**

There is uncertainty about the association between alcohol consumption and stroke, particularly for low-moderate intake. We explored these associations in a large international study.

**Methods:**

INTERSTROKE, a case-control study, is the largest international study of risk factors for acute stroke. Alcohol consumption was self-reported and categorized by drinks/week as low (1–7), moderate (7–14 for females and 7–21 for males), or high (>14 for females and >21 for males). Heavy episodic drinking (HED) was defined as >5 drinks on ≥1 day per month. Multivariable conditional logistic regression was used to determine associations.

**Results:**

We included 12,913 cases and 12,935 controls; 25.0% (n = 6,449) were current drinkers, 16.7% (n = 4,318) former drinkers, and 58.3% (n = 15,076) never drinkers. Current drinkers were younger, male, smokers, active, and with higher-paid occupations. Current drinking was associated with all stroke (OR 1.14; 95% CI 1.04–1.26) and intracerebral hemorrhage (ICH) (OR 1.50, 95% CI 1.21–1.84) but not ischemic stroke (OR 1.06; 95% CI 0.95–1.19). HED pattern was associated with all stroke (OR 1.39; 95% CI 1.21–1.59), ischemic stroke (OR 1.29; 95% CI 1.10–1.51), and ICH (OR 1.76; 95% CI 1.31–2.36). High level of alcohol intake was consistently associated with all stroke, ischemic stroke, and ICH. Moderate intake was associated with all stroke and ICH but not ischemic stroke. Low alcohol intake was not associated with stroke overall, but there were regional differences; low intake was associated with reduced odds of stroke in Western Europe/North America (OR 0.66; 95% CI 0.45–0.96) and increased odds in India (OR 2.18; 95% CI 1.42–3.36) (p-interaction 0.037). Wine consumption was associated with reduced odds of all stroke and ischemic stroke but not ICH. The magnitudes of association were greatest in those without hypertension and current smokers.

**Discussion:**

High and moderate intake were associated with increased odds of stroke, whereas low intake was not associated with stroke. However, there were important regional variations, which may relate to differences in population characteristics of alcohol consumers, types or patterns of consumption.

Stroke is a leading cause of death and disability globally.^1^ Although age-specific incidence appears to be declining in some high-income countries, it is increasing in low- and middle-income countries.^2,3^ Therefore, there is an urgent need to understand the contribution of existing and emerging risk factors for stroke at a population level.^4^

Alcohol use is recognized as a risk factor for a range of diseases.^5^ In particular, heavy episodic drinking (HED) or high intake increases the risk of conditions that are major contributors to a global burden of premature mortality,^5^ including physical injury, cardiovascular disease (CVD), and certain types of cancer. Although light to moderate alcohol use has been associated with a reduced risk of some cardiovascular events,^6-9^ there remains considerable uncertainty for stroke^6^ as apparently protective effects from light or moderate intake may be an artifact of residual confounding.^10^ In addition, mendelian randomization studies^11,12^ and large population cohorts (generally from high-income countries^13,14^) suggest that light-moderate alcohol consumption in mid-life is not associated with a reduced risk of stroke. Given the high global frequency of light-moderate alcohol consumption, it is of considerable relevance to determine whether it is associated with the risk of stroke.

Alcohol use is a complex exposure, with diversity in alcohol products (types and quality) and social context of consumption, which may further vary by region and culture. For example, alcohol intake is an integral part of social life in many regions (e.g., Europe) but discouraged or prohibited in others. Therefore, alcohol consumption as an exposure may represent a multitude of direct and indirect factors, which differ between regions and populations. A limitation of current evidence is that most studies were completed in high-income countries, and there are relatively sparse data for low- and middle-income countries, where alcohol use is increasing^15^ and associations with health may be different.^16^

INTERSTROKE identified that 10 modifiable risk factors were collectively associated with 90% of the global population-attributable risk of stroke.^17^ As INTERSTROKE recruited from a geographically and ethnically diverse population, it is ideally placed to further explore global associations between alcohol intake and stroke.

## Methods

INTERSTROKE is a large international case-control study whose details were published previously.^17^ In brief, cases were defined as patients with first stroke (within 5 days of symptom onset and admitted to hospital within 3 days of presentation) and were recruited from 142 centers in 32 countries between March 2007 and July 2015. Neuroimaging was completed in 99.9% of cases. Information was obtained from the patient or a proxy respondent. Controls, without acute stroke, were recruited from the community or hospital and matched to cases for age (<5 years of difference or <10 years if aged >90 years), sex, and geographic region. We excluded participants from countries where >95% of controls reported never drinking alcohol (Pakistan, Kuwait, Iran, and Saudi Arabia) as responses to the questions on alcohol consumption may not be reliable due to cultural beliefs and social desirability bias.^16^

Risk factors were assessed through standardized structured questionnaires (completed by the participant, proxy, or both) and physical examination. Blood pressure (BP) was measured at the time of interview and estimated preadmission level. Self-reported items included medical history, physical activity, diet (assessed using the Alternate Healthy Eating Index [AHEI]^18^), smoking, and psychological factors.^19^ Hypertension was defined as a self-reported history of hypertension or BP≥140/90 mm Hg (including adjusted admission BP, as previously described^17^). Diabetes mellitus was defined as self-reported history of diabetes or HbA1c ≥ 6.5%. Countries were grouped as follows: (1) Western Europe and North America (Canada, Australia, Germany, Denmark, Sweden, United Kingdom, and Ireland); (2) Eastern and Central Europe (Croatia, Poland, Turkey, and Russia); (3) China; (4) South America (Argentina, Brazil, Chile, Colombia, Ecuador, and Peru); (5) Southeast Asia (Thailand, Philippines, and Malaysia); (6) India; and (7) Africa (South Africa, Mozambique, Uganda, Sudan, and Nigeria).

Alcohol intake was reported as never, former, or current drinker and further characterized^20^ based on total weekly intake as low (1–7 drinks), moderate (7–14 drinks for women or 7–21 drinks for men), or high intake (>14 drinks for women or >21 drinks for men).^16^ HED pattern was defined as >5 drinks in 1 day at least once a month over the previous 12 months. Participants were classified by predominant type of alcohol consumption as (1) beer or other, (2) wine, or (3) spirit or arrack.

All data were transferred to Population Health Research Institute, McMaster University and Hamilton Health Sciences, Canada, for quality control. The study was approved by ethics committees in all centers or countries, and participants (or proxy) provided written informed consent.

### Statistical Analysis

We calculated means and medians to summarize continuous variables, compared by *t* tests or appropriate nonparametric tests. Conditional logistic regression was used to estimate ORs and 95% CIs for all analyses, other than subgroup analysis where we primarily used unconditional logistic regression. Multivariable adjustment included hypertension (yes vs no), smoking (never or former vs current), diet quality (thirds of AHEI), physical activity (inactive vs active), diabetes (yes vs no), cardiac risk factors (yes vs no), lipid levels (thirds of ApoB:ApoA), and stress (little or none vs moderate or severe). Unconditional models were also adjusted for age (continuous), sex (males vs females), and geographic region (7 categories, as previous).

For our primary analyses, we present associations between alcohol intake and all stroke, ischemic stroke, and intracerebral hemorrhage (ICH). For analyses stratified by region or subgroup and all sensitivity analyses, we present associations with all stroke only. We explored whether associations differed by cardiovascular risk factors (hypertension, diabetes, physical activity, diet, and smoking), level of wealth, and education. For analyses by predominant alcohol type, we hypothesized that the associations by predominant alcohol intake would differ between those with and without HED pattern or different levels of intake; therefore, we included these variables on additional adjustment. Differential effects between strata were considered statistically significant if the *p* value for the interaction between the stratifying variable and measure of alcohol intake was <0.05.

As sensitivity analyses, we restricted our sample only to those who completed the questionnaire themselves (to reduce potential bias introduced by proxies). Second, we hypothesized that factors influencing the likelihood that an individual consumes alcohol vary significantly between regions and may be a major source of confounding as these factors are also associated with stroke. Therefore, we completed sensitivity analyses where we calculated a propensity score for current alcohol consumption; drinkers were matched with never drinkers based on this score (i.e., matched on alcohol status rather than case-control). We used logistic regression to predict the probability of current vs never consuming alcohol for every individual in the study (cases and controls), including age, sex, income, occupation, education, geographic region, smoking, hypertension, lipids, diet, physical activity, and diabetes. Participants were matched to nearest neighbor, without replacement, based on propensity to current alcohol consumption. All propensity score–based matching was completed within strata of sex to ensure full matching by sex. Similarly, we also hypothesized that factors influencing the predominant type of alcohol consumed varies between regions and may be a source of confounding. Therefore, we completed analyses where we calculated propensity scores for predominant alcohol type and drinkers were matched with never drinkers (i.e., matched on predominant alcohol type rather than case-control). We used the same methodology as described previously for propensity to current vs never consuming alcohol. Adjusted conditional and unconditional logistic regression are presented for associations between alcohol and all stroke for the propensity score–based matched analyses. Post hoc, we identified that predominant wine drinkers had reduced odds of stroke; therefore, we sought to explore for evidence of a dose-response in the association between absolute intake (number of drinks per week) and odds of stroke, dichotomized by predominant wine vs nonwine drinkers, through restricted cubic splines with 5 knots and unconditional multivariable-adjusted logistic regression. All statistical analyses were performed with Stata/MP 16.1, and a *p* value of <0.05 was considered statistically significant.

### Data Availability

The principal author has full access to the data used in the analyses in this manuscript and takes full responsibility for the data, analyses, and interpretation of findings and has the right to publish these findings. Anonymized data not published within this article may be made available on request from any qualified investigator.

## Results

We include 96% of cases (n = 12,909) and controls (n = 12,934) from INTERSTROKE, whose characteristics were published previously.^17^ Overall, the mean age was 61.8(SD 13.4) years, and 40.5% (n = 10,458) were female (Table 1). Questionnaires were completed by patients (42.5%), proxy (35.8%), or both (21.7%).

**Table 1:**
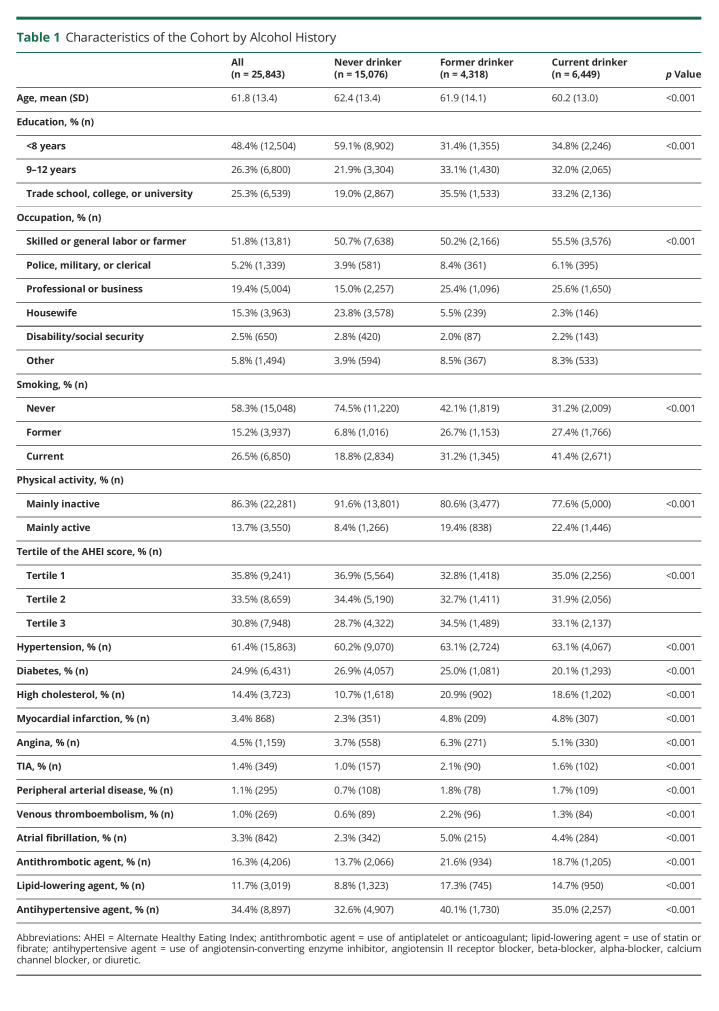
Characteristics of the Cohort by Alcohol History

### Alcohol Intake

**Figure 1:**
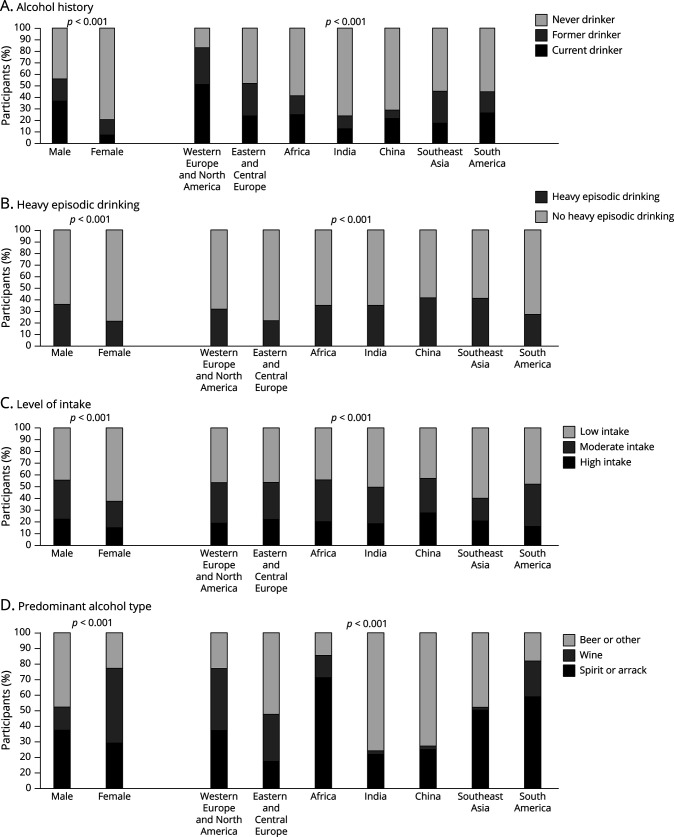
Alcohol Consumption by Sex and Geographic Region (A) Frequency of never, former, and current drinking by sex and region. (B) Frequency of heavy episodic drinking within current drinkers by sex and region. (C) Frequency of low, moderate, and high intake within current drinks by sex and region. (D) Distribution of predominant alcoholic beverage consumed within current drinkers by sex and region.

Overall, 25.0% (n = 6,449) were current drinkers, 16.7% (n = 4,318) former drinkers, and 58.3% (n = 15,076) never drinkers (Table 1). Current drinking was more common in males (*p* < 0.001) and in Western Europe/North America and Australia and South America but least common in India and South East Asia (*p* < 0.001) (Figure 1A). Current drinkers were more likely to be younger, current smokers, physically active, and had higher-paid occupations (Table 1). Never drinkers had the lowest prevalence of hypertension, high cholesterol, previous CVD, and medication use but the highest prevalence of diabetes. Participants with a first stroke had higher cardiovascular risk factor profiles than those without stroke, consistent with previous reports (eTable 1, links.lww.com/WNL/C416). There were significant regional variations in the associations of current drinking and some risk factors, including hypertension, physical activity, and diet quality (eTable 2).

Within current drinkers, HED was more common among males (*p* < 0.001), in China and South East Asia and least common in Eastern and Central Europe (*p* < 0.001) (Figure 1B). Those with HED were younger, more likely to be current smokers, and mainly inactive but less likely to have hypertension, diabetes, high cholesterol, and CVD (eTable 3, links.lww.com/WNL/C416). High intake was more common in males (*p* < 0.001) and in China but least common in Western Europe and North America (*p* < 0.001) (Figure 1C). Those with high intake were younger and more likely to be less educated, current smokers, mainly inactive, and to have hypertension, diabetes, and previous CVD (eTable 3).

Predominant wine consumption was more common in females (*p* < 0.001) and in Western Europe and North America, Eastern and Central Europe, and South America (*p* < 0.001) (Figure 1D). Spirit or arrack consumption was most common in males and in China and India. Wine drinkers were older, more likely to have higher levels of education, professional, or business occupations, and to be mainly active with higher diet quality (eTable 4, links.lww.com/WNL/C416). Spirit or arrack drinkers were more likely to be less educated, current smokers, mainly inactive, and to work as laborers or farmers.

### Alcohol Intake and Stroke

**Figure 2:**
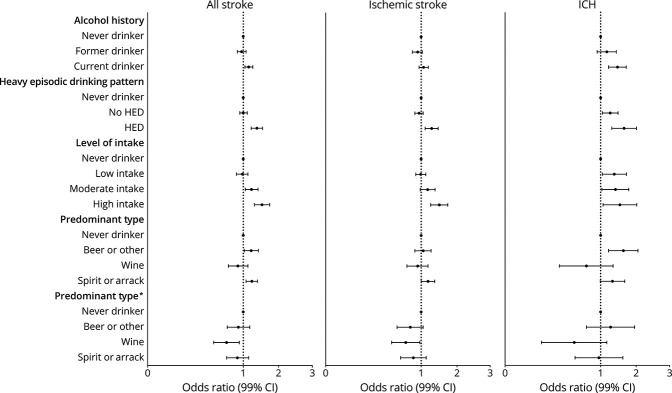
Association Between Alcohol Consumption and Stroke Conditional logistic regression adjusted for hypertension, smoking, AHEI, physical activity, diabetes, cardiac risk factors, ApoB/ApoA, age, stress with pairs matched for age, sex, and region. *Additional adjustment for age, sex, and region. Abbreviation: AEHI = Alternate Healthy Eating Index.

After multivariable adjustment, current drinking was associated with increased odds of all stroke (OR 1.14; 95% CI 1.04–1.26) and ICH (OR 1.50; 95% CI 1.21–1.85) but not associated with ischemic stroke (OR 1.06; 95% CI 0.95–1.19) (Figure 2) (eTable 5, links.lww.com/WNL/C416). There was no association between former drinking and stroke. Within current drinkers, HED was associated with all stroke (OR 1.39; 95% CI 1.21–1.59), ischemic stroke (OR 1.29; 95% CI 1.10–1.51), and ICH (OR 1.76; 95% CI 1.31–2.37). Compared with never drinkers, there was no association between low intake and all stroke or ischemic stroke but increased odds of ICH (OR 1.39, 95% CI 1.04–1.86). High alcohol intake was consistently associated with all stroke (OR 1.57; 95% CI 1.31–1.89), ischemic stroke (OR 1.55; 95% CI 1.26–1.90), and ICH (OR 1.59; 1.06–2.39). Predominant beer or other alcohol was associated with all stroke (OR 1.21; 95% CI 1.03–1.44) and ICH (OR 1.73; 95% CI 1.21–2.46) but not ischemic stroke. Predominant wine consumption was not associated with stroke. Predominant spirit or arrack consumption was associated with increased odds of all stroke (OR 1.23; 95% CI 1.07–1.41) and ischemic stroke (OR 1.18; 95% CI 1.00–1.39) but not ICH. With additional adjustment for HED and level of intake, associations between beer or other alcohol and spirit or arrack consumption and stroke were attenuated, whereas the associations between wine consumption and stroke demonstrated a significantly lower risk for all stroke (OR 0.67; 95% CI 0.49–0.91) and ischemic stroke (OR 0.69; 95% CI 0.49–0.97) but not ICH.

### Alcohol Intake and Stroke by Geographic Region

There were significant differences in the associations of alcohol intake with odds of all stroke by geographic region (Table 2). Current drinking was associated with reduced odds of stroke in Western Europe and North America and increased odds of stroke in India and South America but not associated with stroke in other regions (*p* < 0.001). Among current drinkers, (1) the greatest magnitudes of association between HED and all stroke were seen in South America, Africa, and India (*p* < 0.001); (2) the greatest magnitudes of association between high intake and all stroke were seen in China and South East Asia (*p* = 0.037); and (3) there were no significant differences in the association between predominant alcohol type and all stroke.

**Table 2:**
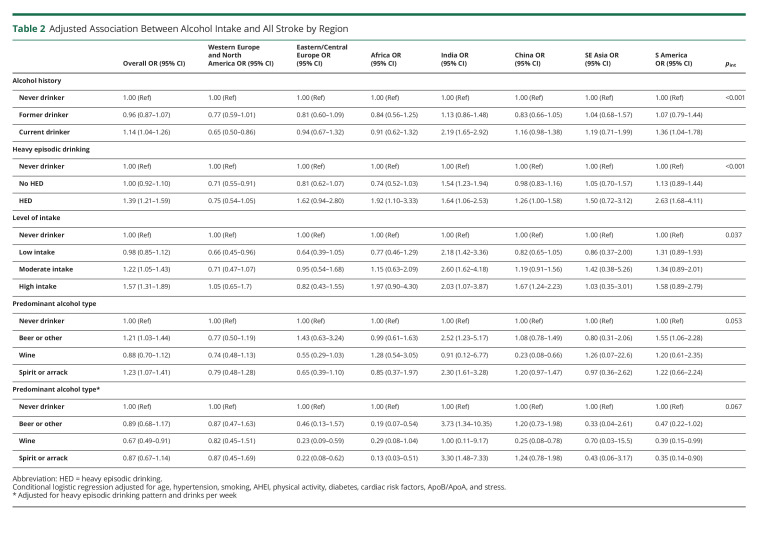
Adjusted Association Between Alcohol Intake and All Stroke by Region

### Subgroup Analyses

The magnitudes of association between all stroke and current drinking, HED, or high intake were greatest in those without hypertension (all *p* = 0.001) and among current smokers (all *p* < 0.001) (eTable 6, links.lww.com/WNL/C416). For current drinking and HED, the magnitudes of association were greatest in those with the lowest tertile of diet quality (both *p* = 0.03). There were no significant differences in associations stratified by physical activity or diabetes. The magnitude of association between current drinking and all stroke was greatest in those with the lowest level of education (*p* = 0.02), but there were no significant differences for HED (*p* = 0.27) or level of intake (*p* = 0.21). There were no differences in the associations between alcohol consumption and all stroke on stratification by thirds of wealth index.

### Sensitivity Analyses

**Figure 3:**
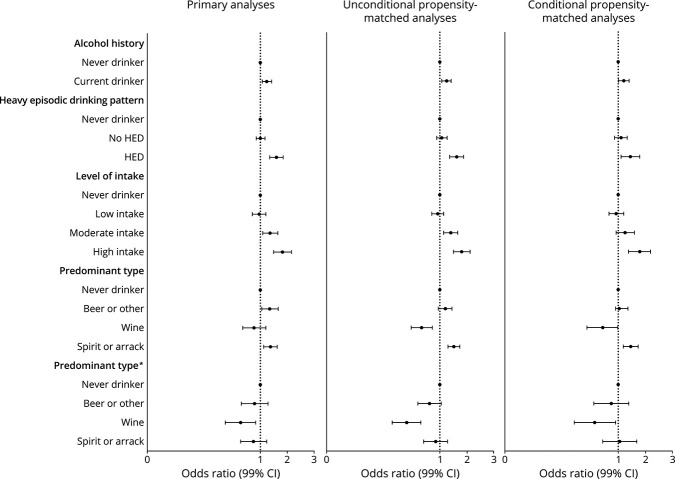
Propensity Score (for Current Drinking) Matched Analyses for the Association Between Alcohol Intake and All Stroke Propensity score for current vs never drinking with adjusted for hypertension, smoking, AHEI, physical activity, diabetes, cardiac risk factors, ApoB/ApoA, and stress. *Additional adjustment for age, sex, and region. Abbreviation: AEHI = Alternate Healthy Eating Index.

Analyses restricted to participants who completed questionnaires themselves (i.e., excluding proxy contributors) showed associations of similar magnitude and direction to our primary analyses, although confidence intervals were wider (eTable 7, links.lww.com/WNL/C416). Propensity score–based matching for current vs never drinking yielded 3,879 matched pairs that were well matched for all factors but sex (12.8% never drinkers vs 14.7% current drinkers were female, *p* = 0.013) (eTable 8). Unadjusted and adjusted conditional logistic regression models show no directional or significant magnitudinal changes in associations between all stroke and current drinking, HED, or level of intake, compared with our primary analyses (Figure 3) (eTable 9). Propensity score–based matching for predominant alcohol type vs never drinking yielded 1,607 matched pairs for beer or other drinking, 709 matched pairs for wine drinkers, and 2,287 pairs for spirit or arrack drinkers, well matched for all measured factors (eTable 10). Associations were largely similar for beer or other drinkers and spirit or arrack drinks, but the magnitude of the association indicating lower risk was more marked for predominant wine drinkers (eTable 11). There was no consistent increase in odds of all stroke by absolute level of intake in predominant wine drinkers but a significant increase in predominant nonwine drinkers (eFigure 1).

## Discussion

In this large international study, we report that current alcohol consumption is associated with all stroke and a greater magnitude of association for ICH after multivariable adjustment. A pattern of HED and high levels of alcohol intake were both associated with increased risk of all stroke, ischemic stroke, and ICH, and we did not observe any reduction in odds of stroke with low alcohol consumption, compared with never drinkers. There were differences in association by the predominant type of alcohol consumed with increased odds of stroke with spirit, arrack, beer, or other alcohol consumption but not wine. Additional adjustment for an HED pattern and level of alcohol consumption yielded a significant reduction in the odds of all stroke and ischemic stroke for predominant wine drinkers.

Our findings are generally consistent with findings from other international studies, including the INTERHEART^6,19^ and PURE studies^16,21^ and previous meta-analyses^14,22-24^ that explored other cardiovascular outcomes in relation to alcohol intake. In general, the conclusions are similar^25^; high alcohol intake was associated with harm, low alcohol intake was associated with little or no protection, and there were complex regional variations. It is hypothesized that alcohol consumption is associated with stroke through multiple mechanisms, including increased BP, alterations in cholesterol, reductions in fibrinogen, altered endothelial function, modulation of inflammation, and provocation of atrial fibrillation or other cardiac arrhythmias.^26^ The main overall associations of alcohol intake and stroke are also consistent with large epidemiologic studies using mendelian randomization approaches. The largest of these studies, involving 161,498 participants, reported that genotype-predicted alcohol intake was associated with a log-linear increase in risk of stroke with predicted alcohol intake >10 units per week, which was higher for ICH than ischemic stroke.^12^ They did not report a lower risk of stroke with low-moderate intake, which contrasts with the results of their conventional epidemiologic analyses that observed a J-shaped association. Our overall findings are more consistent with their mendelian randomization analyses than their traditional epidemiologic approach.

HED and high alcohol were both associated with increases in all stroke, ischemic stroke, and ICH, and magnitudes of association were greatest in those without hypertension and in current smokers. There were significant differences in the characteristics of those who engage in HED or high levels of intake; they were more common in males and were more prevalent in certain regions (e.g., China). Therefore, it is likely that targeted interventions to manage HED or high alcohol intake could be expected to result in consistent benefits across populations but different influences on the absolute incidence of stroke in different regions and between men and women. The associations between alcohol intake and stroke were seen after correcting for other risk factors, suggesting that alcohol intake exerts an effect, separate from any effect on other risk factors, although we cannot exclude residual confounding or additional effects of behavioral, social, or environmental factors.

A wide range of alcoholic drinks were reported by participants, and when we classified individuals by predominant beverage consumed, there were important differences in characteristics between groups. Predominant wine consumption was more common among older individuals, women, in those with higher levels of education and occupation, and most prevalent in Europe and the Americas. This contrasts significantly with predominant spirit or arrack consumption, which was more common in younger individuals, men, lower levels of education and occupation, and most prevalent in China and India. This suggests that there are important societal, cultural, and socioeconomic factors that influence the predominant type of alcohol consumed and current drinking. For all stroke, we report increased odds of all stroke with spirit, arrack, beer, or other alcohol consumption but not wine consumption. In addition, when we also adjusted for HED and level of intake, we found that wine consumption was associated with significantly reduced odds of all stroke and ischemic stroke. Our propensity-matched analyses (for current drinking and predominant alcohol type) showed consistent findings to our primary analyses. It is unclear whether this directionally different association of wine consumption with stroke, compared with other alcohol types, relates to a specific difference in cardiovascular effects of low-moderate consumption of different alcohol types^16,27,28^ or differences in the social and behavioral context of wine consumers (for example, those consuming wine had higher diet scores and higher level of physical activity).

The major strength of this study is that it included a very large study population and was conducted in many countries, in different regions, and involved individuals with different ethnicities.^17^ The case-control design provided a practical approach to achieving the level of diversity that is more representative of global alcohol consumption patterns than studies conducted in a single or limited number of countries in a region. It also includes populations that have previously been excluded. We included a large number of possible covariates to minimize the effects of confounding and used multiple analytic approaches (conditional and unconditional logistic models and propensity-matched analyses), with consistent findings, irrespective of the approach taken. These features increase the generalizability and robustness of our study findings.

Our study has a few potential limitations. First, a case-control design may be potentially open to biases if there is differential recollection of alcohol use between cases and controls, and it may lead to recall bias,^29,30^ where cases may over- or under-estimate alcohol consumption and bias results away from or toward the null, depending on the social or cultural context. This potential source of bias may also be exacerbated in those where the questionnaire was completed by or with the assistance of a proxy. However, sensitivity analyses restricted to only those who completed the questionnaire themselves were consistent with our primary analyses. Selection bias may have resulted from the approach used to identifying controls; this was reduced by excluding controls with a hospital referral diagnosis linked to stroke/TIA and using standardized data collection methods that were applied in the same way to participants with a first stroke and controls. Social desirability bias^31^ may be likely to occur where alcohol use is taboo as respondents may underestimate or underreport consumption, compared with countries where drinking is socially acceptable. Therefore, we excluded countries with the highest prevalence of never drinking in controls. Despite this, the prevalence of current alcohol consumption within controls in INTERSTROKE was lower than estimates from the global burden of disease, although current alcohol consumption does reduce with increasing age.^32^ In common with most observational studies, causality cannot be firmly established. Although we adjusted for multiple confounders and our propensity score analyses were similar to our primary results, the associations observed between alcohol consumption and stroke may be influenced by residual confounding, unmeasured confounders (e.g., genetic differences and variation in alcohol type or preparation), and heterogeneity of social circumstances. Further work is required to explore whether the association between alcohol consumption and stroke is causal, including large cohort studies, as clinical trials are not feasible in this area. Other important limitations are that we were confined to those who survived stroke long enough to reach hospital. We found no association between former alcohol consumption and stroke, perhaps surprising as some individuals stop consuming alcohol due to illness or medical conditions (consistent with the sick quitters hypothesis^33,34^). However, it is also possible that some individuals classified as never drinkers may have stopped drinking for health reasons, leading to an underestimation of associations. Finally, even a large study like INTERSTROKE can have limited power to quantify alcohol effects within some of the countries or regions. Findings in particular geographic regions such as East and Central Europe, Africa, South East Asia, and South America require further exploration within the respective countries.

HED and high alcohol intake were associated with increased odds of all stroke, ischemic stroke, and intracerebral hemorrhage. Although the prevalence of this risk factor may vary by age, sex, and region, the relative increase in odds was consistent across these subgroups. Future initiatives at ensuring healthy lifestyles should include a reduction in high alcohol intake and binge drinking. We did not observe any convincing reduction in stroke risk with low or moderate intake.

## Glossary

AHEI: Alternate Healthy Eating Index
CVD: cardiovascular disease
HED: heavy episodic drinking
ICH: intracerebral hemorrhage
